# Adverse effects of excessive dietary arachidonic acid on survival, PUFA-derived enzymatic and non-enzymatic oxylipins, stress response in rainbow trout fry

**DOI:** 10.1038/s41598-024-63173-x

**Published:** 2024-05-29

**Authors:** Emilie Cardona, Emilien Segret, Cécile Heraud, Jerome Roy, Claire Vigor, Valérie Gros, Guillaume Reversat, Battitte Sancho-Zubeldia, Camille Oger, Anaelle Durbec, Justine Bertrand-Michel, Anne Surget, Jean-Marie Galano, Geneviève Corraze, Yoann Cachelou, Yann Marchand, Thierry Durand, Frederic Cachelou, Sandrine Skiba-Cassy

**Affiliations:** 1 Viviers de Rébénacq, 64260 Rébénacq, France; 2 INRAE, Univ. Pau & Pays Adour, E2S UPPA, NUMEA, 64310 Saint Pée-sur-Nivelle, France; 3 Viviers de Sarrance, 64490 Sarrance, France; 4 Institut des Biomolécules Max Mousseron (IBMM), Pôle Chimie Balard Recherche, UMR5247, CNRS, Université de Montpellier, ENSCM, 34293 Montpellier, France; 5 I2MC, Université de Toulouse, Inserm, Université Toulouse III – Paul Sabatier (UPS), Toulouse, France; 6 MetaboHUB-MetaToul, National Infrastructure of Metabolomics and Fluxomics, 31077 Toulouse, France; 7 Legouessant Aquaculture, 22402 Lamballe, France

**Keywords:** Trout, Arachidonic acid, Oxylipin, Confinement stress, Serotonin, Dopamine, Biochemistry, Physiology

## Abstract

Arachidonic acid (C20: 4n-6, AA) plays a fundamental role in fish physiology, influencing growth, survival and stress resistance. However, imbalances in dietary AA can have detrimental effects on fish health and performance. Optimal AA requirements for rainbow trout have not been established. This study aimed to elucidate the effects of varying dietary AA levels on survival, growth, long-chain polyunsaturated fatty acid (LC-PUFA) biosynthetic capacity, oxylipin profiles, lipid peroxidation, and stress resistance of rainbow trout fry. Over a period of eight weeks, 4000 female rainbow trout fry at the resorptive stage (0.12 g) from their first feeding were fed diets with varying levels of AA (0.6%, 1.1% or 2.5% of total fatty acids) while survival and growth metrics were closely monitored. The dietary trial was followed by an acute confinement stress test. Notably, while the fatty acid profiles of the fish reflected dietary intake, those fed an AA-0.6% diet showed increased expression of elongase5, highlighting their inherent ability to produce LC-PUFAs from C18 PUFAs and suggesting potential AA or docosapentaenoic acid_n-6_ (DPA_n-6_) biosynthesis. However, even with this biosynthetic capacity, the trout fed reduced dietary AA had higher mortality rates. The diet had no effect on final weight (3.38 g on average for the three diets). Conversely, increased dietary AA enhanced eicosanoid production from AA, suggesting potential inflammatory and oxidative consequences. This was further evidenced by an increase in non-enzymatic lipid oxidation metabolites, particularly in the AA-2.5% diet group, which had higher levels of phytoprostanes and isoprostanes, markers of cellular oxidative damage. Importantly, the AA-1.1% diet proved to be particularly beneficial for stress resilience. This was evidenced by higher post-stress turnover rates of serotonin and dopamine, neurotransmitters central to the fish's stress response. In conclusion, a dietary AA intake of 1.1% of total fatty acids appears to promote overall resilience in rainbow trout fry.

## Introduction

As demand for fish and shellfish increases, aquaculture's contribution to the provision of vital nutrients continues to grow. To support the industry in a sustainable way, innovative changes in aquaculture diets, particularly the substitution of fishmeal (FM) and fish oil (FO) with alternative ingredients in aquafeeds, are paramount. The main consumers of FO and FM are carnivorous fish species such as salmonids, including rainbow trout (*Oncorhynchus mykiss*), which contribute significantly to global aquaculture^1^. Despite efforts to replace marine ingredients with plant-based alternatives, full substitution is still suboptimal due to negative effects on fish growth, health, and reproductive efficiency^2,3^.

One of the advantages of FM and FO is that they provide long-chain polyunsaturated fatty acids (LC-PUFAs) from both the n-3 and n-6 series. These PUFAs are synthesized from precursor fatty acids (FAs) that are considered as essential because they must be obtained from dietary sources. These precursors are the linoleic acid (C18:2 n-6, LA) and the alpha-linolenic acid (C18:3 n-3, ALA) for the n-6 and n-3 series, respectively. All fish species require n-6 and n-3 LC-PUFAs, the biologically active forms of which are generally fatty acids with 20 or 22 carbons derived from LA or ALA, namely arachidonic acid (C20:4 n-6, AA) for the n-6 serie and eicosapentaenoic acid (C20:5 n-3, EPA) and docosahexaenoic acid (C22:6 n-3, DHA) for the n-3 serie. While marine fish have a reduced ability to produce PUFAs themselves, different species of freshwater fish have varying abilities to produce PUFAs through biosynthesis and their dietary requirements for PUFAs may vary accordingly^4^. In fish, AA is primarily stored in polar lipids and constitutes a minor component of cell membranes compared to EPA and DHA^5,6^. However, from a functional standpoint, it is the most important n-6 LC-PUFA associated with membrane phospholipids.

For a long time, EPA and DHA were considered to be the only LC-PUFAs that played a major role in fish requirements and AA has received little attention. This historical neglect of AA in fish diets, was partly due to the assumption that the requirement for AA was very low and could be met by the small but significant amounts of AA found in FM and FO^7^. However, it is now recognized that this assumption is too simplistic, as fish may have higher requirements for AA at certain stages of their life cycle, particularly during periods of environmental or physiological stress^8–10^ or during the reproductive phase^11^. AA has been shown to be essential for early larval development in some fish species. For example, AA deficiency can lead to impaired growth and development as observed in gilthead sea bream larvae^12^ and in striped bass larvae^13^. Studies in sea bream have shown that AA supplementation improves growth, survival and immune response in fish subjected to stressful conditions such as metamorphosis, weaning, crowding, grading, and handling^9,14^.

AA is often studied for its implication in the stress response, most commonly attributed to its role as a precursor of enzymatic oxylipins^8,9,12^. Indeed, LC-PUFAs including AA, are precursors of highly bioactive lipid metabolites called oxylipins, which are derived from these FAs via the cyclooxygenase (COX), lipoxygenase (LOX), and cytochrome P450 pathways. Oxylipins act as key mediators in the body's physiological responses^15,16^. Most studies addressing this issue have focused on oxylipins derived from AA, as these are generally considered to be the most abundant and bioactive, whereas those produced from EPA and DHA tend to be less potent^5,17^. In addition, oxylipins derived from AA show stronger anti-inflammatory, vasoconstrictive and proliferative effects than those derived from EPA, although there are some exceptions^15^.

Due to their multiple double bonds, LC-PUFAs are also highly susceptible to reactive oxygen species (ROS). This lipid peroxidation can lead to the formation of various free radical-induced peroxidation products such as F_1_-phytoprostanes (F_1_-PhytoP), F_2_-isoprostanes (F_2_-IsoP), F_3_-isoprostanes (F_3_-IsoP), and F_4_-neuroprostanes (F_4_-NeuroP). These compounds are derived from ALA, AA, EPA and DHA, respectively, and are known as non-enzymatic oxylipins. Recent publications have shown that these metabolites can have several beneficial effects, such as neuroprotection and cardioprotection in humans. They can also be used as markers of oxidative stress^18^.

Currently, the dietary requirements for AA in trout, particularly in fry, are not understood. This lack of knowledge raises concerns, particularly in the context of replacing fishmeal (FM) and fish oil (FO) with alternative ingredients, which could potentially lead to AA deficiency and physiological disorders. Such deficiencies may particularly affect the physiology of trout, especially their response to stress, as has been demonstrated in other fish species. It is therefore essential to determine the specific dietary requirements for AA in this species. This understanding will help to tailor diets to ensure optimal growth, health and performance. In this study, we investigated the effects of varying AA intake on the survival and growth of rainbow trout fry, as well as their capacity for polyunsaturated fatty acid (PUFA) biosynthesis and stress response.

## Methods

### Ethical statements

The experiments were carried out at the Viviers de Sarrance commercial fish farm (Sarrance, France). All fish were bred and handled in strict compliance with the French Ministry of Research (i.e. Decree No. 2013-118) and the European Union guidelines on the protection of animals used for scientific purposes (i.e. Directive 2010/63/EU). The protocol was approved by the Ethics Committee of Aquitaine Poissons-Oiseaux (C2EA-73) and received the approval of the French Ministry of National Education, Research and Innovation (APAFIS approval n° 34404-2021121409143622). The scientists in charge of the experiments received training and personal authorisation. If any clinical signs (i.e. morphological abnormalities, restlessness or uncoordinated movements) were observed, the fish were sedated by immersion in a benzocaine bath (concentration: 20 mg/L) and then euthanised by immersion in another benzocaine bath (concentration: 100 mg/L) for 3 min. The study is reported in accordance with the ARRIVE guidelines (Animal Research: Reporting of In Vivo Experiments).

## Diets

Three experimental diets, which were isonitrogenous, isolipidic, and isoenergetic, were formulated to contain 58.6% crude protein, 10.9% crude lipid, and 22.5 kJ/g. Tables 1 and 2 report the raw materials, proximate composition, and fatty acid composition of the experimental diets. All diets were formulated with Libra® feed formulation software (version 1271; https://www.actemium.be/en/offers/agro/formulation) using the same composition of feed ingredients. They differed only in the source of the oil.

**Table 1:** Raw materials and proximate composition of the three diets.

	For 100 g of pellets	AA-0.6	AA-1.1	AA-2.5
Uncoated pellet	Fish meal (66% protein anchoveta)	9.88		
	Fish meal (70% protein herring)	39.12		
	Marin hydrolysed protein (fish and shrimp by-product)	2.95		
	Squid meal	2.95		
	Krill meal	0.95		
	Fish oil	0.95		
	Wheat gluten	19.66		
	Wheat meal	15.10		
	Amino acids	1.33		
	Additive (vitamin, mineral)	1.87		
	Preservative (antioxidant, antifungal)	0.24		
Oil coated	Sun flower oil	2.6	2.5	2.2
	Omega-3 fish oil-sardine/anchovy (Olvea®, France)	2.2	2.2	2.2
	Omegavie® DHA 400 Algae (Polaris, France)	0.3	0.3	0.3
	Fungi oil (Seanova®)	0.0	0.1	0.3
Nutrient composition	Dry Matter, %	93.3	93.2	93.2
	Crude proteins, % DM	58.6	58.7	58.6
	Lipids, % DM	10.9	10.8	11.0
	Ash, % DM	9.2	9.3	9.3
	Energy, kJ/g DM	22.5	22.6	22.5

Dietary ingredients are expressed on an air-dried basis. Nutrient composition is expressed on a dry matter (DM) basis.

**Table 2:** Main total fatty acids measured in the three experimental diets (% of total FA).

Main fatty acids, % of total FA	AA-0.6	AA-1.1	AA-2.5
Saturated FA sum	22.97	23.15	23.56
Mono-unsaturated FA sum	28.42	28.34	27.66
C18:2 n-6 (LA)	25.22	24.90	23.27
C20:4 n-6 (AA)	0.62	1.06	2.48
C22:5 n-6 (DPA_n-6_)	0.37	0.37	0.37
n-6 Sum	26.60	26.92	26.86
C18:3 n-3 (ALA)	0.92	0.89	0.90
C20:5 n-3 (EPA)	6.61	6.58	6.75
C22:6 n-3 (DHA)	7.53	7.43	7.54
n-3 sum	15.73	15.61	15.89
n-3/n-6	0.67	0.66	0.67

All other measured FAs are presented in the Supplementary Data 1.

The experimental diets named AA-0.6, AA-1.1, and AA-2.5 contained 0.6, 1.1 and 2.5% AA, respectively, but constant levels of EPA and DHA (i.e. approximately 6.6% and 7.5%, respectively). The minimum amount of AA (0.6%) corresponded to the amount of AA supplied by the FM in the extruded pellets, so this diet can be used as a dietary control.

The production of the pellets was carried out in two stages. Firstly, Le Gouessant Aquaculture company produced an extruded but uncoated pellet based on their commercial formulation (NeoSupra AL4, diameter: 1.4 mm; Le Gouessant, Lamballe, Côte d’Armor, Brittany, France). The uncoated pellets were ground to four different mesh sizes (200–400 µm, 400–800 µm, 800–1200 µm, and 1200–1800 µm) at the INRAE experimental facility (Donzacq, France) in order to adapt the pellet size to the growth stage of fry. Secondly, the pellets were manually coated with different amounts of oils (Table 1) to obtain three diets with different levels of AA. Fungal oil (composition: AA oil from the fungus *Mortierella alpina*, sunflower seed oil, vitamin E (dl-α-Tocopherol), ascorbyl palmitate, containing 40% AA; Seanova®) was used as the source of AA.*;* Seanova®) was used as source of AA. It was added at a rate of 0, 0.1, and 0.3% of the diet to achieve the desired AA levels. EPA and DHA were provided in equivalent proportions in the three diets using a fish oil (Olvea® sardine/anchovy oil) and microalgae oil (OmegaVie Algae®, Polaris, France).

### Diet chemical analyses

The chemical analyses of the experimental diets were determined according to AOAC^19^ as follows: Dry matter and ash were determined gravimetrically after drying at 105 °C for 24 h and combustion in a muffle furnace at 550 °C for 16 h according to AOAC^19^ methods 930.15 and 942.05, respectively. Gross energy content was measured using an adiabatic bomb calorimeter (IKA C5003, Heitersheim Gribeimer, Germany). Crude protein was determined by the Kjeldahl method^20^.

### Growth trial

Prior to the experiment, eyed eggs from a commercial farm (Viviers de Sarrance) were stored in a 400 L basin at the experimental site. The eggs were kept in an open water circuit at 16 °C ± 2 °C. After hatching, fry were kept under the same conditions until the yolk sacs were resorbed, which took about 2 weeks. After the yolk sacs were resorbed, fry were counted and divided into 20 batches of 200 fish each. These batches were placed in 8-L racks (20 × 20 × 20 cm), which were themselves positioned in 400-L basins.

After two weeks of rearing, fry were transferred to 30-L racks, which were also positioned in 400-L basins. A flow rate of 300 L per hour was maintained in each basin, allowing the water to be completely renewed every 30 min.

During the growth period, the groups were manually fed different diets (n = 4 replicates per diet). The fish were fed until the first feed refusal, 5 times a day during the first week and then 4 times a day for the remaining weeks. The biomass of each tank was weighed every 2 weeks to monitor growth. Dead fish were removed each daily. At the end of the experiment, the fish were counted to calculate survival, and 30 fish per tank were weighed to calculate mean individual body weight. Weight gain and daily feed intake were calculated as follows:

$${\text{Weight}}\;{\text{gain}} = \, \left( {{\text{final}}\,{\text{body}}\,{\text{weight}} - {\text{initial}}\;{\text{body}}\;{\text{weight}}} \right) \, \times {1}00.$$

$${\text{Daily}}\;{\text{Feed}}\;{\text{intake}} = { 1}00 \times {\text{WI}}/\left( {{\text{days}} \times \left( {\left( {{\text{FBio}} + {\text{IBio}}} \right)/{2}} \right)} \right)$$

In this formula, IBio and FBio represent the initial and final fish biomass, respectively (in g). WI is the wet intake, i.e. the total amount of wet feed distributed during the trial (in grams). Days is the total duration of the experiment in days.

### Sampling and stress challenge

The sampled fish were first sedated by immersion in an iso-eugenol solution at a concentration of 20 mg/L and then euthanized by immersion in an iso-eugenol solution at a concentration of 100 mg/L for 3 min.

To study the stress response of the fish, two different stressors were applied during rearing. After 7 weeks of rearing, some fish from each treatment were submitted to a 48-h fasting period, which is considered a moderate stressor for juvenile fish^21^. One week later, other fish were subjected to a confinement challenge, which is considered a more acute stressor for the fish. The confinement challenge protocol was adapted from^22^. For this confinement challenge, all fish from each rack were grouped together in a single tank for 10 min in order to increase the density from 18.6 to 140 kg/m^3^. From a practical point of view, the fish were transferred to 8-L racks (20 × 20 × 20 cm), which were placed in the 400-L tanks. This arrangement allowed for constant water renewal without disturbing the oxygen concentration. To achieve the confinement density in these 8-L racks, the volume of water in the 400-L tanks was carefully adjusted, so that each rack contained only 4 L of water. After 10 min of this confinement, the fish were immediately returned to their original environment, restoring the more accommodating density of 18.6 kg/m^3^. They were then allowed to recover for 40 min before sampling.

After each of these two stresses, two whole fish per tank (n = 8 per diet) were sampled, frozen in liquid nitrogen and stored at − 80 °C until further analysis for measurement of monoaminergic neurotransmitters (serotonin and dopamine).

Twenty additional fish per diet, from those fasted for 48 h, were collected for analysis of the FA profile and plasma serotonin and dopamine levels. Samples were taken from fasted animals to avoid interference from residual feed in the stomach and intestines that could alter fatty acid profiles The remaining fish were fed and, six hours after the meal, when lipid metabolism peaks, 8 whole fish from each treatment were euthanized and sampled. These fish were frozen in liquid nitrogen and stored at − 80 °C for subsequent studies, i.e. analysis of the expression of genes involved in the endogenous biosynthesis of PUFAs and characterization and quantification of enzymatic and non-enzymatic oxylipins. Each fish was individually minced under liquid nitrogen and multiple aliquots were taken for each fish and stored at − 80 °C for future analysis. The aim was to perform different analyses on the same fish individuals.

**Figure 1:**
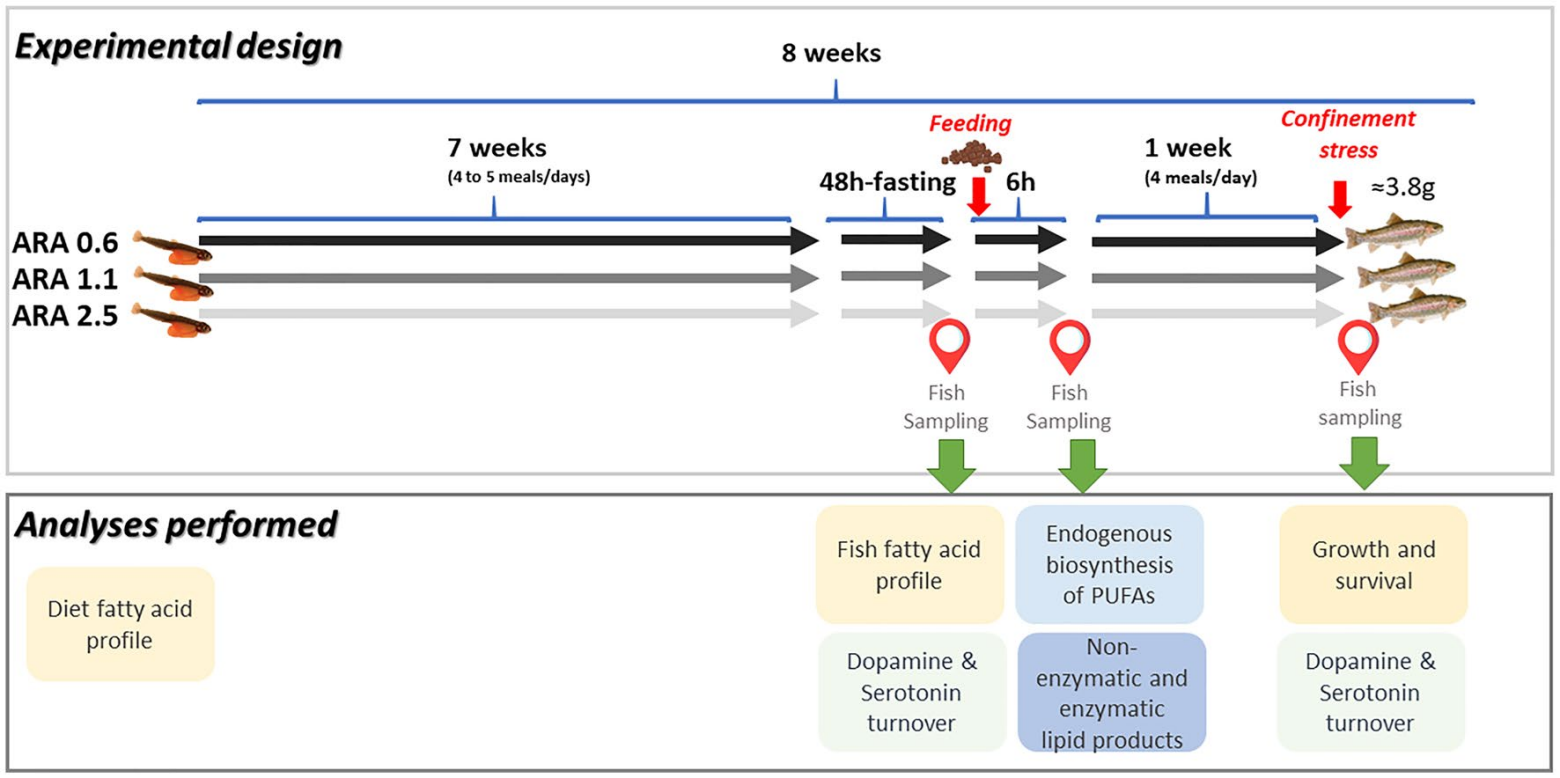
Overview of experimental design and analyses performed.

Figure 1 summarizes the experimental design.

### Fatty acid analysis

The lipid content and fatty acid profile of diets and fish were analyzed. The method has previously been described in Cardona et al.^23^. Briefly, total lipids were extracted according to Folch et al.^24^ with dichloromethane/methanol (MeOH) (2:1, v/v), containing 0.01% (m/v) of butylated hydroxytoluene (BHT) as antioxidant. The percentage of lipids was determined by gravimetry. Fatty acid methyl esters (FAME) were prepared according to Shantha and Ackman^25^. FAME were analyzed through GC-FID (Varian 3900). The FAs were identified by comparing their retention times with a known standard mixture (Sigma, St Louis, MO, USA) and peaks were integrated using Varian Star Chromatography Software (Star Software (version 6.41; URL: https://www.agilent.com/en/product/gas-chromatography/gc-systems). The results for individual FA were expressed as percentage of total identified FA methyl esters (FAME).

### Molecular analysis

RNA extractions were performed on whole fish (n = 8 per treatment). The method was previously described by^23^. Briefly, grounded fish were homogenised in Trizol reagent (Ambion) at a ratio of 1 mL per 100 mg of tissue using Precellys®24 (Bertin Technologies) and total RNA was extracted according to the manufacturer's instructions. The quantity and quality of RNA was assessed by measuring its absorbance at 260 and 280 nm using a Nanodrop 1000 spectrophotometer (Thermo Scientific) in conjunction with NanoDrop ND-1000 software (version 3.7, https://nanodrop-nd-1000.software.informer.com). A step of reverse transcription to cDNA was performed using the super script RNAse H-reverse transcriptase kit (Invitrogen) with random primers (Promega, Charbonnières, France). Luciferase control RNA (Promega) was added to each sample at the start of reverse transcriptase. Genes involved in the biosynthesis of long-chain fatty acids, in particular those coding for the elongation of very long chain fatty acid proteins 2 and 5 (elovl2 and elovl5) and fatty acid desaturase 2 (fads) and 6 (fads6), were analysed by quantitative RT-PCR (Roche Lightcycler 480 system). The data were then normalised to exogenous luciferase transcript abundance. Primer sequences are detailed in Sup data 3.

### Enzymatic oxylipins

To extract enzymatic oxylipins, the individual fry were crushed using a FastPrep®-24 Instrument (MP Biomedical) in 1 mL of HBSS (Invitrogen). After two crush cycles (6.5 m/s, 30 s each), 1000 µL of the homogenate (approximately 15 mg of tissue) were collected and subsequently mixed with cold MeOH (300 µL). Internal standard (5 µL of Deuterium-labeled compounds) was added. After centrifugation at 900×*g* for 15 min at 4 °C, the supernatants were transferred to 2 mL 96-well deep plates and diluted with H_2_O to a total volume of 2 mL. Subsequently, samples underwent solid phase extraction using an OASIS HLB 96-well plate (30 mg/well, Waters), which had been conditioned with MeOH (1 mL) and equilibrated with 10% MeOH (1 mL). Following sample application, the extraction plate was washed with 10% MeOH (1 mL). After drying under aspiration, lipid mediators were eluted with 1 mL of MeOH. Before LC–MS/MS analysis, samples were evaporated under nitrogen gas and reconstituted in 10 µL of MeOH.

LC–MS/MS analyses of oxylipins were carried out as previously described^26^. In brief, enzymatic lipid mediators were separated on a Zorbax SB-C18 column (2.1 × 50 mm, 1.8 µm) (Agilent Technologies) using an Agilent 1290 Infinity HPLC system (Technologies) coupled to an ESI-triple quadrupole G6460 mass spectrometer (Agilent Technologies). Data were acquired in Multiple Reaction Monitoring (MRM) mode with optimized conditions for ion optics and collision energy. Peak detection, integration, and quantitative analysis were performed using Mass Hunter Quantitative analysis software (version: 12.1; https://www.agilent.com/en/product/software-informatics/mass-spectrometry-software/data-analysis/quantitative-analysis, Agilent Technologies), relying on calibration lines established with commercially available oxylipin standards (Cayman Chemicals).

### Non-enzymatic oxylipins

Following a lipid extraction, levels of non-enzymatic oxylipins in the fry were quantified using micro-LC–MS/MS^27^. In a nutshell, the samples underwent a lipid extraction step (Folch extraction) with the addition of Internal Standards, followed by alkaline hydrolysis. Subsequently, the metabolites were concentrated through a solid phase extraction process using weak-anion exchange materials. The concentrated mediators were then subjected to analysis by micro-LC–MS/MS. The mass spectrometry analyses were conducted on a Sciex QTRAP 5500 (Sciex Applied Biosystems) with electrospray ionization (ESI) in negative mode. Fragmentation ion products from each deprotonated molecule [M–H]^–^ were detected in the multiple reaction-monitoring mode (MRM). Concentrations of non-enzymatic lipid mediators were determined using calibration curves based on the area ratio of analytes to IS. The data were processed using the MultiQuant 3.0 software (version 3.0; https://sciex.com/products/software/multiquant-software, Sciex Applied Biosystems).

### Monoaminergic neurotransmitters: serotonin and dopamine

Each fish sample was homogenized using a Precellys® tissue homogenizer in a buffer containing 50 mM phosphate and 1 mM EDTA (pH 6.5 ± 0.05). Following the initial centrifugation (14,000×*g* for 20 min at 4 °C), the supernatant was deproteinized using an equal volume of 10% metaphosphoric acid (MPA) solution. After a second centrifugation (14,000×*g* for 5 min at 4 °C), the supernatant underwent filtration using a 0.22 µm poly-vinylidene di-fluoride unit (Millipore, Bedford, MA, USA). Subsequently a third and final centrifugation (14,000×*g* for 5 min at 4 °C) was performed. The resulting metabolite mixtures were stored at − 20 °C and used within a month for liquid chromatography to fluorescence analysis. Catecholamines (dopamine pathway) and indolamines (serotonin pathway) from both standard mixtures and polar metabolite extracts were separated on Luna PFP (2) column (150 × 4.6 mm, 3 μm; Phenomenex) using a Waters® Acquity H-Class Plus UHPLC System. This system was equipped with a thermostatted autosampler and supported by a Waters® Acquity Multi-λ Fluorescence Detector (Milford, MA, USA). The Waters® Empower™ Pro 3 software managed data acquisition and quantification. Chromatographic separation occurred on a Phenomenex® Luna PFP (2) column (150 × 4.6 mm i.d. 3 μm) maintained at 30 °C (Torrance, CA, USA). The system's injection volume was 10 μL, and a flow rate of 0.4 mL/min was maintained. A binary solvent system, comprising (A) pH 4.3 ± 0.05 10 mM phosphate buffer and (B) methanol, was utilized. This mobile phase had undergone filtration through in-line 0.2 μm membrane filters. The elution pattern was as follows: 15 to 20% (B) from 0 to 11 min, maintained at 20% (B) for 3 min, 20 to 30% (B) from 14 to 17 min, 30 to 60% (B) between 17 to 17.1 min, held at 60% (B) until 22 min, 60 to 15% (B) from 22 to 22.1 min, and finally, 15% (B) held for 7.9 min for column equilibration. Double excitation/emission fluorescence detection monitored the eluate: 285 nm/355 nm for 5-HT and 5-HIAA; 228 nm/306 nm for L-DOPA and HVA.

The metabolites of interest were identified by matching the retention times with those of standards. Quantification relied on the integration of peak areas, compared against standard calibration curves (with R^2 correlation > 0.999) for each metabolite. These curves were linear, ranging from 0.01 to approximately 3 pmol/injection for 5-HT, 5-HIAA, L-DOPA, and HVA. Data normalization was based on protein concentrations, measured using a BCA Kit (Interchim) according to the manufacturer's instructions on the homogenate collected prior to MPA extraction step. The Waters® Empower™ Pro 3 software (version FR4; https://www.waters.com/waters/en_US/Empower-Chromatography-Data-System-%28CDS%29/nav.htm?cid=10190669&lset=1&locale=en_US&changedCountry=Y) managed data acquisition and quantification.

### Statistical analysis

Results are expressed as means and standard deviations. A statistical analysis of the data was carried out using R studio software (version 4.0; https://www.r-studio.com/fr/)^28^. The percentage data were subjected to an arcsine square root transformation to stabilise the variance and improve the suitability for statistical analysis. A Kruskal–Wallis test evaluated the effect of diet on fish mortality rate, weight gain and daily feed intake. One-way ANOVA was used to assess the impact of dietary treatment on individual body weight, fish FA profile, relative gene expression, and enzymatic and non-enzymatic oxylipin concentrations. The adequacy of the final linear model was assessed by making residual plots to check the normality. These analyses did not reveal abnormalities, and thus the analyses were validated. A Tukey post-hoc test was performed to differentiate the treatments.

## Results

### Growth and survival

Dietary AA level did not affect growth, weight gain and daily feed intake but the lowest level of AA (AA-0.6) resulted in significantly higher mortality, although the percentage of mortality was very low (1.6% for the AA-0.6 diet compared to 0.7% for the other diets, p-value = 0.001; Table 3). The confinement stress did not cause mortality.

**Table 3:** Effect of AA dietary level in diet on fish performances.

	AA0.6	AA1.1	AA2.05	*p*-value
Mortality rate, %	1.83 ± 0.43^a^	0.67 ± 0.27^b^	0.67 ± 0.27^b^	0.001
Final body weight, g	3.37 ± 0.22	3.28 ± 0.15	3.49 ± 0.33	n.s
Weight gain, %	338.08 ± 21.85	316.03 ± 15.30	336.85 ± 33.03	n.s
Daily feed intake, %	3.00 ± 0.25	3.12 ± 0.14	2.94 ± 0.38	n.s

Results are expressed as means ± standard deviations. One-way analysis of variance was carried out in order to assess effects of diet on individual final weight; replicates correspond to different individual fingerlings (n = 120 fingerlings per treatment). “n.s”: not significant Results are expressed as means and standard deviations. Different letters indicate significant differences between groups, which were investigated with a Tukey post hoc test. Weight gain = (final body weight − initial body weight) × 100. Daily Feed intake = 100 × WI/(days × ((FBio + IBio)/2)). (IBio and FBio represent the initial and final biomass, respectively (in g). WI is the wet intake, i.e. the total amount of wet feed distributed during the trial (in g). Days is the total duration of the experiment in days).

### Fatty acid profile of diets and fish

The proportion of the main fatty acid as a percentage of total fatty acids in the diets and fish is presented in Tables 2 and 4, respectively. The profiles of all the fatty acids in the 3 experimental diets and fish are available in the supplementary data (Sup data 1 & 2).

**Table 4:** Main total fatty acids (% of total FA) and lipid content (% dry matter (DM)) in fry according to diets.

Main fatty acids, % of total FA	AA-0.6	AA-1.1	AA-2.5	*p*-value
Saturated FA sum	24.90 ± 0.17^a^	25.26 ± 0.45^ab^	25.59 ± 0.50^b^	0.009
Mono-unsaturated FA sum	30.09 ± 0.29	29.96 ± 0.43	29.89 ± 0.24	n.s
C18:2 n-6 (LA)	19.00 ± 0.18^a^	18.59 ± 0.22^b^	17.77 ± 0.20^c^	1.93e10^–10^
C20:4 n-6 (AA)	0.93 ± 0.04^a^	1.15 ± 0.03^b^	1.84 ± 0.03^c^	2.21e10^–16^
C22:5 n-6 (DPA_n-6_)	0.39 ± 0.01^a^	0.42 ± 0.02^b^	0.47 ± 0.01^c^	1.49e^10–8^
n-6 Sum	22.89 ± 0.15	22.74 ± 0.27	22.75 ± 0.32	n.s
C18:3 n-3 (ALA)	0.77 ± 0.01	0.76 ± 0.02	0.77 ± 0.01	n.s
C20:5 n-3 (EPA)	3.06 ± 0.04	3.02 ± 0.10	3.08 ± 0.21	n.s
C22:6 n-3 (DHA)	11.45 ± 0.14	11.44 ± 0.21	11.34 ± 0.19	n.s
n-3 sum	16.21 ± 0.16	16.15 ± 0.32	16.05 ± 0.30	n.s
n-3 / n-6	0.77 ± 0.01	0.77 ± 0.01	0.77 ± 0.01a	n.s
Lipids, % DM	28.9 ± 1.00	29.4 ± 1.61	29.3 ± 1.08	n.s

Results are expressed as means ± standard deviations. One-way analysis of variance was carried out in order to assess effects of diets on fatty acid proportion (n = 8 pools of 20 fry per treatment). Different letters indicate significant differences between groups, which were investigated with a Tukey post hoc test. All other measured FAs are presented in the Supplementary Data 2.

The fatty acid profiles of the three diets were notably similar, with the main difference being the AA content, which distinguishes the three diets. The levels of n-3 PUFAs remained constant across the diets. The increase in AA was accompanied by a proportional decrease in LA, so that the total n-6 fatty acid content remained constant in all diets.

The differences in LA proportions in the diet are reflected in the fish. The proportion of AA in the fish was found to be influenced by the amount of AA in the diet. A similar profile was observed between the diet and the fish, with a higher proportion of AA in the fish whose diets contained more AA. However, the variations in AA proportions were less pronounced in the fish than in the respective diets. In the diets, the percentage variation in AA content between the AA-0.6 and AA-1.1 diets was + 71%, while it increased to 300% between the AA-1.1 and AA-2.5 diets. In contrast, in the fish, the percentage variation in AA was + 24% between the AA-0.6 and AA-1.1 diets and + 98% between the AA-1.1 and AA-2.5 diets. The fish also had significantly different DPA levels, even though the diets had the same DPA content. The DPA content was highest in the AA-2.5 diet, intermediate in the AA-1.1 diet and lowest in the AA-0.6 diet.

The proportions of EPA and DHA were the same in all three diets and in the fish. The ratio of n-3 to n-6 FAs was similar in fish fed the three diets.

### PUFA endogenous biosynthesis

Expression of *fads2*, *fads6* and *elovl2* genes was not affected by diets. The mRNA expression of *elovl5* was higher in fish fed the AA-0.6 diet compared those fed the other two diets (Table 5).

**Table 5:** Relative expression of genes involved in PUFA biosynthesis metabolism in fish according to diets.

Genes	AA-0.6	AA-1.1	AA-2.5	*p*-value
*fads2*	0.98 ± 0.30	0.89 ± 0.41	0.98 ± 0.55	n.s
*fads6*	0.64 ± 0.29	0.63 ± 0.32	0.74 ± 0.26	n.s
*elovl5*	1.31 ± 0.75^a^	0.59 ± 0.26^b^	0.70 ± 0.27^b^	0.01
*elovl2*	1.24 ± 0.43	0.74 ± 0.29	1.05 ± 0.52	n.s

Results are expressed as means ± standard deviations. One-way analysis of variance was carried out in order to assess effects of diet. Replicates correspond to individual fish (n = 8 per treatment). “n.s”: not significate; *p-value < 0.05; **p-value < 0.01; ***p-value < 0.001. Different letters indicate significant differences between groups, which were investigated with a Tukey post hoc test. (Elovl: elongation of very long chain fatty acids proteins; fads: fatty acid desaturase).

### Enzymatic oxylipins

**Figure 2:**
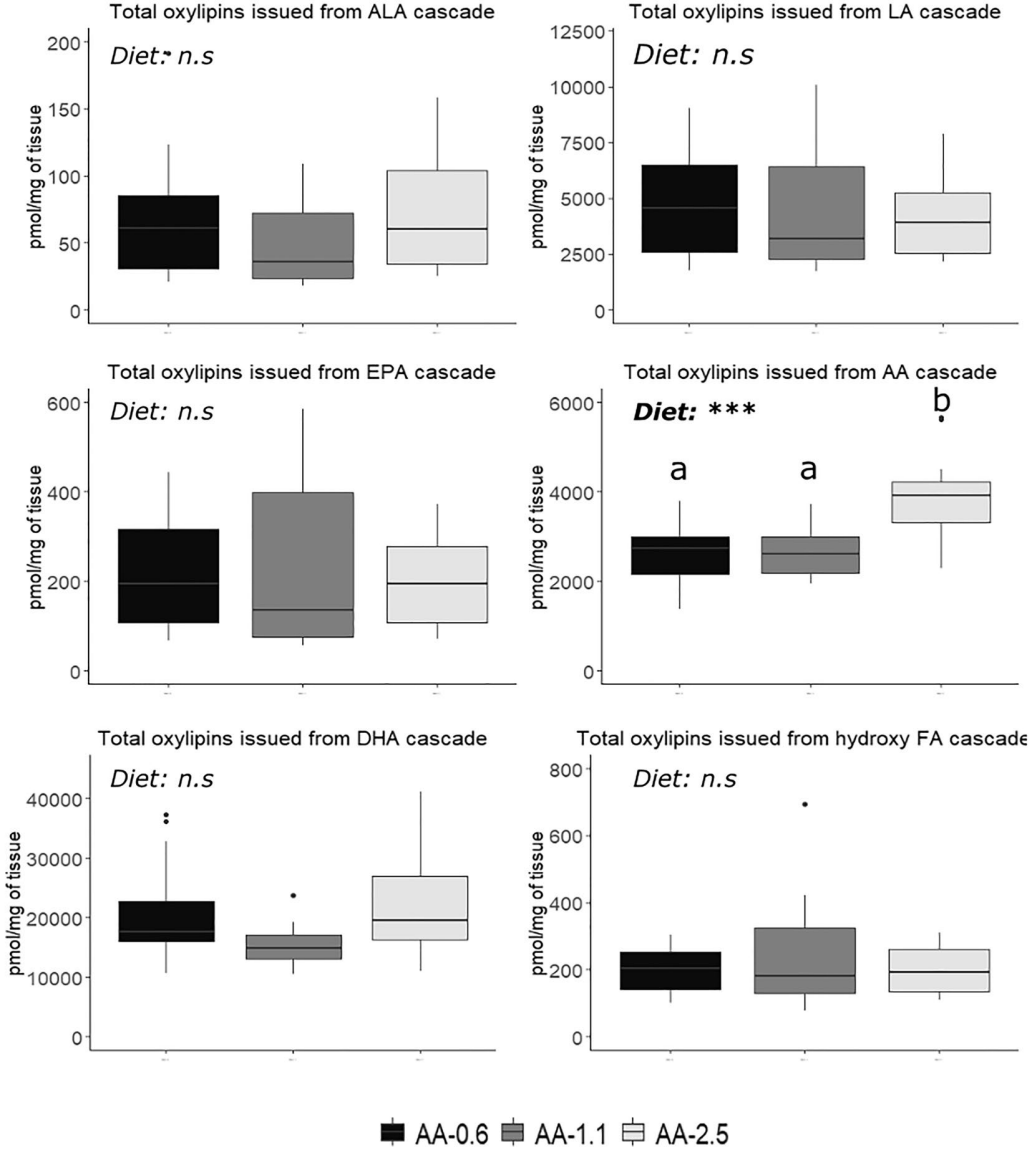
Free oxylipins derived from each PUFA cascade (pg/mg of tissue) in fish 6-h post-feeding according to diets. One-way analysis of variance was carried out in order to assess effects of diet. Replicates correspond to individual fish (n = 6 per treatment). Different letters indicate significant differences between groups, which were investigated with a Tukey post hoc test.

The oxylipins derived from each PUFA cascade were measured in whole fish 6 h after feeding (Fig. 2). Only AA-derived oxylipins were affected by the diet.

The AA-derived oxylipins were overproduced with the AA-2.5 diet compared to the other two diets. A detailed analysis of the different AA-derived oxylipins revealed that the main components contributing to this overproduction were three hydroxyeicosatetraenoic acids (8,12, and 15-HETE), two prostaglandins (PGD2 and PGE2) and one thromboxane (TXB2) (Table 6).

**Table 6:** Free oxylipins from arachidonic acid (AA) (pg/mg of tissue) in fish 6-h post-feeding according to diets.

Name	AA-0.6	AA-1.1	AA-2.5	*p*-value
5-HETE	51.10 ± 7.96	58.32 ± 16.58	62.57 ± 10.58	n.s
8-HETE	128.69 ± 39.35^a^	139.65 ± 35.83^a^	221.17 ± 68.71^b^	0.001
12-HETE	1852.57 ± 562.80^a^	1854.92 ± 404.43^a^	3016.68 ± 1034.73^b^	0.002
15-HETE	97.51 ± 60.44^ab^	55.89 ± 17.7^a^	128.48 ± 105.07^b^	0.016
5-oxo-ETE	3.59 ± 5.58	5.18 ± 5.11	7.10 ± 8.19	n.s
8-isoPGA2	3.02 ± 2.79	8.53 ± 12.42	5.55 ± 3.45	n.s
PGD2	5.36 ± 1.92^a^	3.95 ± 2.29^a^	10.36 ± 6.87^b^	2e10^–4^
PGE2	125.65 ± 38.94^a^	149.56 ± 56.98^ab^	208.48 ± 77.70^b^	0.016
PGF2α	3.38 ± 0.71	6.54 ± 8.36	5.50 ± 1.71	n.s
15dPGJ2	0.27 ± 0.42	0.93 ± 1.49	0.39 ± 0.43	n.s
TXB2	5.04 ± 2.16	4.58 ± 2.69	6.64 ± 2.72	0.043

Results are expressed as means ± standard deviations. One-way analysis of variance was carried out in order to assess effects of diet. Replicates correspond to individual fish (n = 6 per treatment). “n.s”: not significate. Different letters indicate significant differences between groups, which were investigated with a Tukey post hoc test. (*HETE* hydroxyeicosatetraenoic acid, *PG* prostaglandins, *TBX* thromboxane).

### Non-enzymatic oxylipins

Non-enzymatic oxylipins were measured in whole fish 6 h after feeding (Table 7).

**Table 7:** Non-enzymatic lipid oxidation metabolites (pg/mg of tissue) in fish 6-h post-feeding according to diets.

Parent FA	Name	AA-0.6	AA-1.1	AA-2.5	*p*-value
ALA	ent-16-*epi*-16-F_1t_-PhytoP	3.69 ± 0.71^ab^	3.19 ± 1.07^a^	4.80 ± 0.83^b^	0.018
	ent-16-F_1t_-PhytoP	3.14 ± 0.27	3.14 ± 0.87	3.99 ± 0.65	n.s
	9-*epi*-9-F_1t_-PhytoP	3.41 ± 0.65^ab^	2.99 ± 0.99^a^	4.31 ± 0.84^b^	0.04
	16-B_1t_-PhytoP	6.99 ± 0.92^b^	5.14 ± 0.87^a^	8.62 ± 1.26^c^	0.0001
	9-L_1t_-PhytoP	6.37 ± 1.33^ab^	4.12 ± 0.84^a^	7.84 ± 1.79^b^	0.001
EPA	18(*R*)-18-F_3t_-IsoP	49.95 ± 13.81	42.23 ± 14.08	55.96 ± 5.95	n.s
	18(*S*)-18-F_3t_-IsoP	21.29 ± 5.34	18.81 ± 6.97	24.94 ± 2.58	n.s
	8(*S*)-8-F_3t_-IsoP	6.52 ± 1.66	6.20 ± 1.91	7.19 ± 0.55	n.s
	8(*R*)-8-F_3t_-IsoP	6.61 ± 1.59	6.13 ± 1.66	7.27 ± 0.55	n.s
	5(*S*)-5-F_3t_-IsoP	114.32 ± 39.38	92.83 ± 38.10	130.48 ± 30.61	n.s
	5(*R*)-5-F_3t_-IsoP	224.54 ± 74.45	182.90 ± 70.73	263.35 ± 60.15	n.s
AA	15-*epi*-15-F_2t_-IsoP	4.20 ± 0.94^a^	3.62 ± 0.97^a^	6.68 ± 0.94^b^	0.0001
	15-F_2t_-IsoP	3.18 ± 0.64^a^	2.73 ± 0.79^a^	5.00 ± 1.28^b^	0.002
	5(*R* + *S*)-5-F_2t_-IsoP	11.21 ± 2.98^a^	8.94 ± 3.82^a^	20.28 ± 5.92^b^	0.001
	5F_2c-_IsoP	22.47 ± 4.81^a^	18.03 ± 5.57^a^	34.89 ± 7.31^b^	0.0006
	15(*R*)-PGF2	3.64 ± 0.85^a^	2.99 ± 0.89^a^	5.68 ± 0.89^b^	0.0002
	α PGF2	21.31 ± 3.67^a^	20.71 ± 2.64^a^	30.08 ± 7.62^b^	0.01
DPA	14-F_3t_-NeuroP	2.19 ± 0.55	1.88 ± 0.54	2.41 ± 0.46	n.s
	4(*R* + *S*)-4-F_3t_-NeuroP	3.28 ± 0.40^ab^	2.71 ± 0.56^a^	4.13 ± 0.96^b^	0.008
DHA	13A(*RS*)-13-F_4t_-NeuroP	8.76 ± 3.96	4.76 ± 1.08	8.20 ± 4.16	n.s
	13B(*RS*)-13-_F4t_-NeuroP	5.56 ± 2.64	2.99 ± 0.68	5.03 ± 2.88	n.s
	20(*R*)-20-F_4t_-NeuroP	6.64 ± 1.85	4.73 ± 1.12	7.35 ± 2.89	n.s
	20(*S*)-20-F_4t_-NeuroP	4.79 ± 2.01	3.59 ± 0.95	4.43 ± 0.89	n.s
	10(*S*)-10-F_4t_-NeuroP	8.14 ± 2.30	6.69 ± 1.73	7.66 ± 1.45	n.s
	10(*R*)-10-F_4t_-NeuroP	10.72 ± 2.74	9.21 ± 2.64	9.98 ± 1.62	n.s
	14(*R*)-14-F_4t_-NeuroP	2.56 ± 0.46	2.12 ± 0.54	2.54 ± 0.45	n.s
	14(*S*)-14-F_4t_-NeuroP	3.04 ± 0.74	2.17 ± 0.42	2.94 ± 0.73	n.s
	4(*RS*)-4-F_4t_-NeuroP	12.47 ± 3.61^ab^	8.07 ± 2.29^a^	13.53 ± 4.32^b^	0.038

Results are expressed as means ± standard deviations. One-way analysis of variance was carried out in order to assess effects of diet. Replicates correspond to individual fingerlings (n = 6 per treatment). “n.s”: not significate. Different letters indicate significant differences between groups, which were investigated with a Tukey post hoc test. (*PhytoP* phytoprostane, *IsoP* isoprostane, *PG* prostaglandin, *NeuroP* neuroprostanes).

The dietary effect was most pronounced on the non-enzymatic oxylipins from ALA and AA. Of the 5 metabolites detected from ALA, 4 phytoprostanes, namely ent-16-*epi*-16-F_1t_-PhytoP, 9-*epi*-9-F_1t_-PhytoP, 16-B_1t_-PhytoP, and 9-L_1t_-PhytoP, showed increased production in fish fed the AA-2.5 diet compared with those fed the other two diets. Similarly, the 6 metabolites from AA identified namely 4 isoprostanes (15-*epi*-15-F_2t_-IsoP, 15-F_2t_-IsoP, 5(*RS*)-5-F_2t_-IsoP, 5-F_2c_-IsoP) and 2 prostaglandins (15(*R*)-PGF2α and PGF2α) were more abundant when using the AA-2.5 diet compared to the other two diets.

On the other hand, the production of these metabolites from EPA and DHA was little or not affected by the different diets. Of the 15 metabolites studied, only one, the 4(*RS*)-4-F_4t_-NeuroP derived from DHA, was less produced with the AA-1.1 diet than with the AA-0.6 diet.

### Serotoninergic and dopaminergic turnover in response to 48 h-fasting and confinement stress

**Figure 3:**
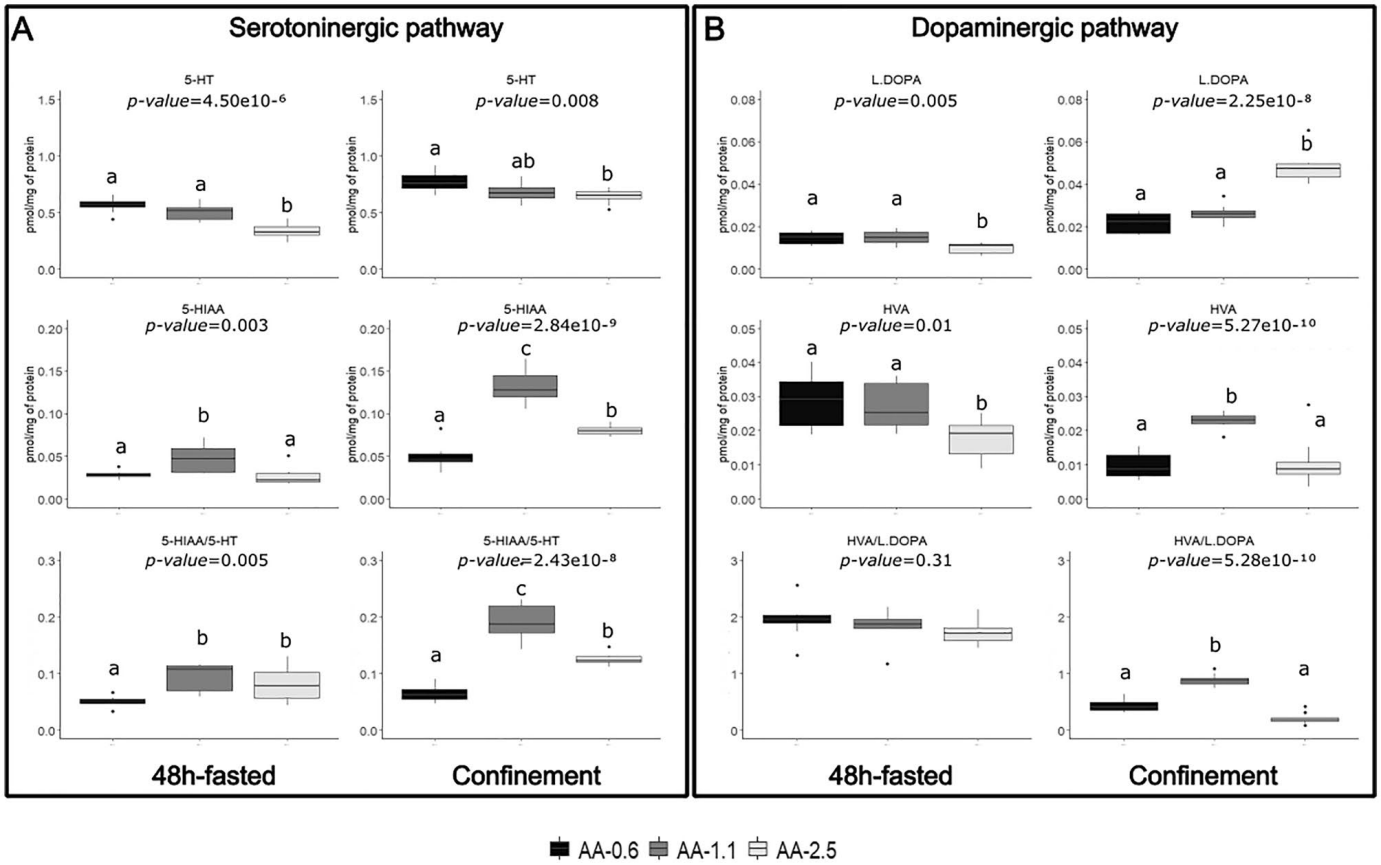
Fish concentration of monoaminergic neurotransmitters according to diets after 48-h fasted and confinement stress (pmol/mg of protein) (**A**) serotonin and metabolite concentrations (5-HT; 5-HIAA and 5-HIAA/5-HT ratio) and (**B**) dopamine precursor and metabolite concentrations (L-DOPA; HVA and HVA/LDOPA ratio). One-way analysis of variance was carried out in order to assess effects of diet. Replicates correspond to individual fingerlings (n = 8 per treatment). Different letters indicate significant differences between groups, which were investigated with a Tukey post hoc test. *5-HT* 5-hydroxytryptamine, *5-HIAA* 5-hydroxyindoleacetic acid, *HVA* Homovanillic acid, *L.DOPA* L-3,4-dihydroxyphenylalanine. *n.s*. not significate.

Figure 3 shows the serotoninergic and dopaminergic levels in response to 48 h fasting and confinement stress, through the production, degradation, and ratios according to the diets provided. After 48 h of fasting, trout fed the AA-2.5 diet exhibited lower serotonin (5-HT) levels, resulting in reduced 5-HT degradation product (5-hydroxyindole acetic acid, 5-HIAA). In contrast, trout fed the AA-0.6 diet had similar 5-HT levels to those fed the AA-1.1 diet but showed a reduced ability to degrade 5-HT, as indicated by significantly lower 5-HIAA concentrations compared to the AA-1.1 diet. These findings led to a significantly lower 5-HIAA/5-HT ratio for the AA-0.6 diet, indicating reduced serotoninergic activity.

Following a confinement stress, trout fed the AA-2.5 diet had lower 5-HT levels compared to the AA-0.6 diet. However, 5-HT was less degraded to 5-HIAA with both the AA-2.5 and AA-0.6 diets when compared to the AA-1.1 diet, which showed the highest level of 5-HIAA. Consequently, these results led to a significantly lower 5-HIAA/5-HT ratio for both the AA-0.6 and AA-2.5 diets compared to the AA-1.1 diet, indicating reduced serotoninergic activity.

Concerning the dopamine pathway, following a 48-h fasting period, trout fed the AA-2.5 diet showed a lower level of the dopamine precursor (L-3,4-dihydroxyphenylalanine; L-DOPA) compared to those on the AA-0.6 and AA-1.1 diets. Additionally, trout fed the AA-2.5 diet showed a decreased level of the dopamine degradation product, namely homovanillic acid (HVA), in comparison to the AA-0.6 and AA-1.1 diets. Notably, there was no significant difference observed in the HVA/L-DOPA ratio.

After confinement stress, despite a higher production of L-DOPA with the AA-2.5 diet, L-DOPA was less degraded to HVA. On the other hand, a significantly higher degradation of L-DOPA was observed in the AA-1.1 diet compared to the other two diets. This resulted in a significantly higher HVA/L-DOPA ratio with the AA-1.1 diet, indicating a higher dopaminergic activity.

## Discussion

The FA composition of fish broadly reflects that of the diet, supporting results reported in previous studies^29,30^. While the content of AA in fish increases with higher dietary AA, which is similar to reports in other fish species^31–33^, the rate at which fish tissues increased was lower than the rate at which diet increased. Indeed, the conversion rates differ significantly, as evidenced by increases of + 71% and + 300% increase between AA-0.6 and AA-1.1, and AA-1.1 and AA-2.5 diets, respectively, while in fish, the increase is only + 24% and + 98%. Furthermore, despite similar proportions DPA_n-6_ in the diets, different levels of DPA_n-6_ levels were observed in the fish, with higher DPA_n-6_ corresponding to higher dietary AA. This suggesting a differential conversion of AA to DPA_n-6_ depending on the dietary AA level. The synthesis of DPA from AA involves 2 successive elongation steps, followed by a desaturation and finally a beta-oxidation. The gene expression of *elovl5*, known for its role in fatty acid elongation, is significantly higher with the AA-0.6 treatment, suggesting a more efficient conversion of AA to DPA when dietary AA is low. Given that *elovl5* is involved in the biosynthesis of both n-6 and n-3 PUFAs, and considering that n-3 fatty acids such as EPA and DHA remain constant in the fish, it appears that only the n-6 biosynthesis pathway is affected. This suggests that AA is utilized and converted differently depending on its level in the diet^33,34^. However, the results show that fish mortality is significantly higher when low levels of AA are provided in the diet. Similar results have been reported in sea bream larvae, where a positive correlation was found between increasing dietary AA levels and survival^9,12^.

AA is converted by a number of enzymes to produce several active by-products, known as oxylipins (i.e. eicosanoids = oxylipins derived from AA), each with its own unique role. The results underline a pronounced influence of dietary AA intake on the eicosanoids derived from AA in rainbow trout, specifically highlighting that the AA-2.5 diet led to increased eicosanoid production from AA. These findings are consistent with those from other fish species that also showed increased levels of eicosanoids from AA in various tissues when fed a higher AA diet^31,35–38^. Despite the potential interplay of AA with EPA and DHA in membrane phospholipid integration, this study did not observe any significant influence of diets on EPA and DHA oxylipin levels. This lack of influence may be correlated with equivalent levels present in both the food and the fish. In our study, increased dietary AA led to increased synthesis of several forms of HETE, including 5-, 8- and 12-HETE, and PGs in the fish. Excessive AA-derived oxylipins, particularly HETE and PGs, are known to induce inflammation^17^. While inflammation can be beneficial in regulated situations, such as wound healing or fighting infection, it can be detrimental if left uncontrolled^38^. In fish, there's evidence that excessive consumption of n-6 PUFA can increase inflammation and lead to negative outcomes, such as triggering oxidative stress^36,38^. In addition, Koven et al.^9^ suggested that excess dietary AA in fish could disrupt the balance of cellular fatty acids, inhibit growth and induce inflammation. These results suggest that there is an overproduction of oxylipins, some of which have inflammatory properties, when a very high AA diet is consumed. This type of diet could therefore have a detrimental effect on the health and performance of the animals.

Notably, fish fed the AA-2.5 diet showed a marked overproduction of non-enzymatic oxylipins derived from AA and ALA. In contrast, the metabolites of EPA and DHA were either not affected or only slightly affected by dietary AA levels. Non-enzymatic oxylipins, often used as indicators of oxidative stress^18^, indicate a cellular environment flooded with reactive oxygen species (ROS). In this study, the AA-2.5 treatment, resulted in an overproduction of several F_2_-IsoPs, F_1_-PhytoPs and α PGF2 compared to the other two treatments. An increase in F_2_-IsoPs and F_1_-PhytoP is known to be associated with cellular oxidative damage^39^. In particular, F_2_-IsoPs are used as a marker of lipid peroxidation in inflammatory diseases^40^. In mammals, excess dietary AA has been shown to potentially increase ROS production, leading to oxidative stress^41^. In fish, AA is known to be an endogenous source of ROS^42^. All this information suggests that the AA-2.5 diet contains too much AA, which could lead to oxidative cell damage and inflammation.

To understand the wider implications of these results, we need to consider the cumulative effects of the increase in all AA-derived metabolites, i.e. oxylipins. This class of compounds, derived mainly from AA in the AA-2.5 group, may together promote the development of an environment that is not only inflammatory but also oxidative. In line with our findings, Bao et al. also found, that a high dietary AA intake in *Acanthopagrus schlegelii* led to the overproduction of eicosanoids, which subsequently triggered oxidative stress, as evidenced by the measurement of malondialdehyde levels, a marker of general lipid peroxidation. This situation can damage cellular structures, disrupt physiological processes and compromise the overall ability of fish to resist external threats.

While dietary AA deficiency appears to increase mortality in rainbow trout, the study also looked more closely at how different dietary AA concentrations and its metabolites affected their resilience to two specific stressors: a 48-h fasting period and confinement stress. Indeed, changes in dietary PUFAs, including n-3 and n-6, and the relationship between them, could potentially modify the stress response of fish through altered eicosanoid production^8,9,12,36,43^. Several studies have shown that oxylipins are involved in the control of osmoregulatory processes and the regulation of the stress-induced hypothalamic-pituitary-interrenal axis, which facilitates the release of cortisol, the major corticosteroid in teleost fish^44–48^.

Neurotransmitters are emerging as sensitive indicators of stress in animals, as both serotonergic and dopaminergic systems are rapidly activated in several cases of acute and also chronic stress^49–52^. Numerous mammalian studies suggest that dietary FA can modulate the central stress axis, particularly through effects on serotonin and dopamine neurotransmission^53,54^. Given the importance of these neurotransmitters in stress modulation and their key role in the hypothalamic-pituitary-internal axis^52^, they are considered essential for stress adaptation in fish and mammals^55^. However, how FA composition directly influences stress adaptation in fish, and in particular the turnover of serotonin and dopamine, remains an area of active research. This study showed that, under confinement stress, the conversion of 5-HT and L-DOPA to their respective metabolites (5-HIAA and HVA) was increased in trout fed the AA-1.1 diet compared to those fed the AA-0.6 and AA-2.5 diets, resulting in higher 5-HT and L-DOPA turnover rates. Imbalances, such as insufficient synthesis or degradation, can affect fish health and survival^56^. For both serotonin and dopamine turnover, the ratio of the tissue concentration of their metabolites to that of the parent monoamine is often used as an index of neural activity. A higher ratio indicates a more efficient stress-response mechanism, which underlines a better adaptation to stress in rats^57,58^ and fish^59^. In this study, neurotransmitter turnover appears to be optimal with a diet containing 1.1% AA, making the fish more resilient to stress. Observational data further supports the potential relationship between dietary fatty acid composition, particularly AA levels, and the efficacy of the stress response in fish.

## Conclusion

Based on our results, it appears that a dietary AA level of 1.1% is the most appropriate for rainbow trout fry compared to levels of 0.6% and 2.5%. The AA-1.1 diet resulted in minimised mortality, balanced neurochemical responses to stress and harmonious production of AA-derived metabolites. Taken together, these results suggest that the AA-1.1 diet may provide a more balanced nutritional profile for the well-being and robustness of trout during their critical early life stages.

### Supplementary Information

Supplementary Legends.

Supplementary Information 1.

Supplementary Information 2.

Supplementary Information 3.

## Data Availability

The data supporting the findings of this study will be openly available in a public repository at https://entrepot.recherche.data.gouv.fr with the DOI link 10.57745/WONKOF.
