# Supplementary material for: Adverse effects of excessive dietary arachidonic acid on survival, PUFA-derived enzymatic and non-enzymatic oxylipins, stress response in rainbow trout fry
Source: Sci Rep. 2024 May 29;14:12376. doi: 10.1038/s41598-024-63173-x (PMC11137042; doi:10.1038/s41598-024-63173-x)
Supplement: Supplementary file 1 — Supplementary Legends. [file 41598_2024_63173_MOESM1_ESM.docx]

**List of supplementary data**

**Sup data 1:** Total fatty acid profiles (% of total FA) of the three experimental diets.

**Sup data 2:** Total fatty acids (% of total FA) and lipid content (% dry matter (DM)) in fry according to diets.

Sup data 3: Sequences of primer pairs used for gene expression analysis by qRT-PCR.
